# The Proceedings

**Published:** 1852-07

**Authors:** 


### DENTAL REGISTER OF THE WEST.

Vol. V. JULY, 1852. No. 4.

### Selections.

### THE PROCEEDINGS

*Of the twelfth Annual Meeting of the American Society of Dental Surgeons, held in Philadelphia on the* 5*th*, 6*th, and* 7*th of August*, 1851.

(Continued from page 181.)

Dr. White.—*Mr. President*, I would wish to say a few words in defence of arsenic, though not as used, to overcome the necessity of excising the nerve, for that I believe is the object of all. I have not the least doubt that every gentleman who has removed the pulp of a tooth at all, has had much more success by so doing, than if he had destroyed its *vitality* by any agent, and left it remain in the tooth. But it is desirable to get the nerve out with the least possible pain; therefore, the question arises, is there anything that will take away its sensibility, but will not injure the tooth after the pulp is out. If the tooth in other respects is not injured, the next question is, can arsenic be applied in any form with safety to the future health of the tooth? I answer that I believe it can—and that conclusion is drawn from the experience of the use of arsenic for thirteen years with an earnest desire to arrive at the truth. In a series of papers by me on the subject, but which, perhaps, have not been seen by those present, there were reasons given why the pulp of the tooth should have its vitality destroyed previous to its excision; but I stated that I preferred the actual cautery, where it could be properly applied—but it could not be applied successfully in many teeth. One reason that I preferred in some cases Arsenious Acid for the destruction of the nerve, was that it made a more deep and deadly slough than any agent I know of. By the use of it, the pulp can be taken out and produce less pain in the operation, than by the use of any other substance. If you apply enough to destroy the vitality, two-thirds of the way down to the apex of the root, you do not injure the external membranes any more at the apex than by the ordinary cautery. The only question is, does it act laterally so as to produce irritation of the external membranes. I answer that it does not. The bony structure does not take up the arsenious preparation as rapidly as the nerve does; and if it is in a proper condition to be absorbed, it will destroy the vitality of the pulp in a very short time—say in twelve hours—in twenty-four hours at the farthest—and in that time if it is not sufficient to destroy the pulp, how can it injure the bony structure. The whole object is to destroy the vitality of the pulp, and if it will destroy it in six hours, it should then be removed; but repeated experience proves that it must be left in a longer time. If a small portion has permeated the bony structure and caused periosteal inflammation, it is as likely to subside as though it had been caused by any other substance. We see cases where the gums and cheek are swollen to an enormous size, and yet it subsides, and the tooth, if plugged, will remain comfortable for years. Another reason I gave was, that if it could be performed without pain to the patient, it did not render them timid or prevent them from having a nerve destroyed again, as it saves him all the pain that is possible. In hundreds of cases where the operator has attempted to take the nerve out, and the patient knowing it, would never go to the office again. Many persons have said to me, "I would never go to such a dentist, because he tortured such an one so much." Now, arsenious acid is a means of preventing this pain, and there is no danger where sufficient arsenious acid does not remain in the tooth to destroy the vitality of the periosteal membrane. The whole fault as spoken of, has resulted more from ignorance. Operators have been induced to believe that they could put in any quantity, and let it be in as long as they pleased. But it is not so. It is asserted in the United States Dispensatory that the more rapidly an arsenious paste unites with, and a sloughing of the parts produced, the less extensively is it absorbed.

It has been the habit to apply arsenic dry—what is there that you can apply that is less soluble than dry arsenic? Arsenious acid should be ground with creosote until it is impalpable, and as it is largely soluble in that substance, it is in the best possible condition to unite speedily with the pulp. If the vitality of the pulp is destroyed as far as two-thirds of the way down to the apex of the root, it can be removed better, than if the vitality had not been obtunded—and I rarely think of any other method than by applying the acid and letting it remain for twelve or twenty-four hours. It should be observed that the more porous the condition of the tooth, the less time the acid should be left in, though it will permeate the pulp of one tooth as well as of another. I do not apply arsenious acid down the root of the tooth, lest it should injure the external membrane.

The only question is, can arsenic be put in a tooth without necessarily involving its utter destruction? I say it can. On the second of July I applied arsenious paste to a cavity which opened to the pulp on the posterior part of an incisor of a boy of nine years of age. On the fourth, I filled that tooth in the root two-thirds of its depth at least, and it presents as much vital appearance now, as its neighbor—and I have treated cases in that way long ago, which now present the same appearance. In '42 and '43, I prepared a thesis for the degree of Doctor of Medicine, on this subject; and I gave a list of one hundred cases which I had watched for two years, and in eighty-four of the cases, there was no pain in destroying the pulp of the tooth. I found it to vary with the temperament of the patient.

My experience is, that where the nerve has been inflamed, it will be the least likely to give pain when the paste is applied. If the operation is performed in the evening, it will be more likely to give pain, but if performed in the cool of the morning, it will not be likely to give pain in one case out of twenty, I will guarantee—but if it is in the evening, it will give pain in nine cases out of ten. A recently exposed nerve gives more pain than one that has been frequently inflamed.

I have no objection to the use of the cautery—I have no preference for arsenic, except that it is the most deadly poison I can lay my hands on;—if you can give me something that will do it faster—I will take one share in that stock. The great object is to manipulate without giving pain, and I find that I give less pain by using the arsenious acid. As to filling the tooth as soon as the pulp is taken away, I do not do it—but I wait to see how far I have destroyed the vitality of the pulp.—Nature, besides, is better calculated to excise the pulp at the point where it is most necessary, than any instrument we can apply. If we fill a tooth that has been bleeding but a few minutes, the anastomosing *blood vessels* do not at once take back all the blood that returned previously through the pulp, and that function cannot be established without engorging the blood-vessels around the root of that tooth; and it is better to wait till that condition of the blood-vessels has passed off, before filling the tooth; and then you have a better opportunity of saving the tooth, when all the foreign matter is removed, such as the fragments of dead pulp and blood-clot. I have had cases where it was filled immediately, and the patient had returned the next day with so great congestion that the tooth had to be extracted at once. I contend that the necessity of waiting till the anastomosing circulation is established, is one of the most important points in the treatment of the teeth, and there is a greater degree of certainty in waiting a few days than in filling the tooth immediately.

The principal point for which I rose, was to give my evidence that I could destroy the vitality of the pulp with less pain by using arsenic than by any other means, and without endangering the health of the tooth.

Dr. Westcott.—I would like to ask a question or two of Dr. White for information. Do I understand you to say that you are not particular about destroying the nerve to the apex of the tooth?

Dr. White.—No, I am not.

Dr. Westcott.—Did you say that it was the absorption of the arsenic that destroyed it?

Dr. White.—I have said it was the absorption.

Dr. Westcott.—If it is the absorption, and has a progression upwards, what guarantee have you that it is not carried through the apex?

Dr. White.—I have none.

Dr. Westcott.—What guarantee have you that it will stop at any given point, and if it goes through, is there not a possibility of its producing mischief?

Dr. White.—It will produce mischief if it goes through—but I never destroy the pulp entirely—I do not pass the arsenic low down in the root, for fear of its going through. I use other substances to get rid of the balance of the pulp.

Dr. Westcott.—Supposing you have destroyed the pulp two-thirds of the way to its apex, and you decide to fill the tooth, have you any special means or rule by which you stop your filling at a certain point, so as not to crowd upon the portion of nerve still remaining?

Dr. White.—When I am using a small probe to remove the pulp, I pass it as low down as I can—it being large enough to be handled—I cannot get quite to the apex, except in one or two classes of the teeth—and I think, that getting to the apex is rather more of a beau ideal of what treatment ought to be.—I never travelled to the apex, without it is a very large root.—I said two-thirds, because I know I never can get to the apex of the root—it is as fine as a hair for a third of its length.

Dr. Westcott.—The point is, what means have you of knowing whether the arsenic has not been carried clear through to the apex, and there made it liable to inflammation?

Dr. White.—I would answer that I have none, except the inflamed state of the root of the tooth, and I think if it does, the part can as well recover from that corrosive agent as from any other. But I do not mean to say that inflammation invariably ensues—I deny that it is always carried through, and the only symptoms I have that it is not, is that it don't cause inflammation afterwards. I would state that I was enabled to follow those one hundred cases for two years, in which time I extracted six of the teeth for alveolar abscess—but a great number I am enabled to watch yet, and there has not been any alveolar abscess.

Dr. Arthur.—I also have been very much interested in this matter for a good many years past. I have always used arsenic in some form for destroying the vitality of the pulp before removing it, and still use it with satisfactory results. On first entering the profession, I applied it as well as I knew how, but with such bad consequences that I ceased to make use of it.—I am indebted to Dr. White, for hints which induced me again to turn my attention to its use, and for seven or eight years past have employed it with great satisfaction. My principal object in rising at this time, is to call the attention of the Association to a new agent for destroying the vitality of the pulp, or rather an old agent in a new form. For about a year, at the suggestion of a gentleman from Cincinnati, who then stated that it had been used in the west, a long time for this purpose, I have been using the ore of cobalt. This, it is well known, contains about fifty per cent. of pure arsenious acid, which, indeed, is principally procured from this ore. I prefer the arsenic as it exists in cobalt, for the purpose of destroying the vitality of the pulp, for the simple reason that its effects in my hands have been more satisfactory. I use the simple pulverized ore, by saturating a small piece of cotton with creosote, and taking upon it about the twentieth part of a grain. I never apply it except I am satisfied that the pulp is fully exposed. I generally make two applications, which in most cases, I find quite sufficient for the purpose. If I am satisfied that the case coming into my hand, requires the extirpation of the pulp, I apply the cobalt without subjecting the patient to the pain of removing the decayed bone from the cavity. I cover it with cotton saturated in an alcoholic solution of gum sandrach, which, with very light pressure—a very important matter—can be made to retain its place. This I allow to remain twenty-four hours.—On removing it, I am able very fully to expose the pulp. I then apply the cobalt directly to the surface of the pulp, secure it as above directed, and allow it to remain a week. At the end of this time it will generally be feasible to remove the pulp to the very extremity of the root, with but slight pain to the patient. Before adopting this practice, to which I was a long time coming, under the influence of the great dread of the effects of arsenic upon the peridental membrane with which I was infected, I had a great deal of trouble in these cases where patients would not bear firmly the pain attending the removal of the remains of the pulp from the fang. I found it easy enough to destroy the vitality of the pulp to the opening of the fang, and a little way up, but then in consequence of the difficulty of bringing any thing into direct contact with the remaining sensitive part, I was generally compelled to remove it without regard to the pain inflicted, which was sometimes exceedingly severe. I was generally in the habit of making several applications of the arsenic, before I used cobalt in this way, and as Dr. Maynard's practice has been alluded to in this connection, I may state that I know that this was also, and I think is now his practice. I have never allowed arsenic to remain so long as the cobalt, and am therefore unable to say whether it could be done with safety. I should hesitate to try it on account of its solubility.

Dr. Westcott has just stated that in any case where arsenic is applied to the sensitive surface of a cavity of decay, it is certain to destroy the vitality of the tooth. This remark has a particular bearing upon a paper published by me in a late number of the "American Journal of Dental Science," in which I contend and endeavor to show that this can be done, with advantage. We all know, and I know well, that arsenic applied to the bone of a tooth, even when but slightly decayed, and allowed to remain long enough, will effect the destruction of the pulp; but that it will do so in all cases, when allowed to remain only long enough to remove that great tenderness of the bone, which is often found so serious an obstacle to good operations, is not the fact—I know that it is not so.

Dr. Westcott.—I will be more explicit on the point—I meant that so far as I have had a chance of observing, these teeth which have been treated with arsenic in the usual way, with the view of destroying the sensibility of the bony structure, I have usually found worked bad.

Dr. Arthur.—May I ask what is meant by the usual way.

Dr. Westcott—Allowing it to remain twelve or twenty-four hours, four days, or a week, as has been stated in this discussion.

Dr. Arthur.—No allusion, as far as I am aware, has been made in this discussion, to the use of arsenic for removing the sensibility of the dentine, but for the entire destruction of the vitality of the pulp, which is quite another matter.

Dr. Westcott.—I incidentally stated that in my general experience, I had seen much mischief from the use of arsenic—as it had killed the teeth of several—destroying their sensibility.

Dr. Arthur.—The same sweeping assertion, that arsenic could not in any case be applied for this purpose, without eventually destroying the vitality of the tooth so treated, has been frequently made, and I think it ought to be met with a hearty denial of its truth.

There is another important point to be considered in connection with this matter of destroying the nerve, and that is the inflammation of the investing membrane of the root, which is very liable to occur, in the course of the operation, whether arsenic is used to destroy the sensibility of the pulp or not.—The best means of treating this inflammation, when it presents itself, is a subject of very great importance, as the successful preservation of many teeth depends upon it. It is impossible here to enter fully upon this subject, and I now allude to it merely to call attention to the topical use of chloroform for the purpose. I have found its effects more satisfactory in many cases than any agent known to me. Whether it acts as a sedative or counter-irritant, I am unable to say. I generally apply it, or direct its application by wetting the finger with chloroform and touching the gum repeatedly along the course of the root or roots of the tooth under treatment, or saturating a small piece of cotton with it, and laying it against the affected part. Since using chloroform for this purpose, I find that it has been used in France as a topical application in cases of neuralgia with alleged success. About two months ago, a gentleman called upon me to ascertain if I could do any thing to relieve him of pain which he had been suffering for several weeks, with one of his lower molar teeth. He supposed the nerve would have to be destroyed. On examination I found that the nerve was already dead, and that he was suffering from inflammation of the peridental membrane. I advised the application of chloric ether. He called in a few days to say that in five minutes after he applied it, he was relieved of pain, and had since been quite free from it. I then removed the remains of the pulp, filled the roots firmly, and the cavity in the crown, a day or two afterwards. Some weeks after this I saw him again, and there had been no recurrence of the painful symptoms.

Dr. Bridges.—I would remark that this subject was introduced by me, with no unkindness whatever to Dr. Westcott.

A remark has been made which reminds me of a course of practice pursued by a friend of mine. He has endeavored to preserve the nerve alive by cutting it off above the wounded part, taking out the wounded part, and after medical treatment filling the tooth. He told me that in the last two or three years he had taken out fillings to see the state of the teeth, and that in several cases a deposit of white foam had been found in the nerve cavity, and a beautiful healthy state of the nerve had taken place. If this is a fact, it is worthy of notice; and I believe that if one half of the attention was paid to keeping the nerve alive that is paid to killing it, there would be found some remedy that would save almost every nerve. I recollect when I was a boy, in going across the mountains of Vermont, the horses ran away and a boy had his leg mashed to pieces; a young doctor was sent for, and he said send to my father's for the instruments and have his leg cut off, but an old doctor in the neighborhood, whom the young one called an old *granny* said, let us try to plaster it up; this was done, and in three or four weeks the boy had a better leg than a wooden one, and afterwards had a good leg. And I propose now that we try to save instead of trying to kill. The nerves are all very much alike, and if you wound a nerve in almost any other place it may be brought back to a healthy condition. Why cannot a nerve in the tooth be made alive again?

What I told that Dr. Westcott said was literally true. I took it at the time very unkindly, because I thought he meant me; for I used to kill the nerve and fill the tooth over it, but it was a species of quackery.

Dr. Robertson.—The general run of discussion seems to have been on the second aphorism. And it seems to indicate that there is no other treatment except the destruction of the exposed nerve. In my own practice for the last two years in a great many cases where the nerve was not inflamed, but where I had exposed it in preparing the tooth for filling, I have used a small quantity of collodion, which makes a smooth coat over the exposed nerve, and I have filled on that with almost uniform success. And it seems to me that it is better than destroying the nerve. I have practiced it for two years with scarcely a failure that I know of. It seems to me that these aphorisms should be worded somewhat in accordance with the suggestion of Dr. Westcott.

President.—I would explain that we have now under consideration the best mode by which a nerve, when it becomes necessary may be destroyed. We may take up the best mode of preserving it afterwards.

Dr. Robertson.—This is why I object to it, because it would seem to imply that that is the only mode of treatment.— On motion adjourned.

---

#### AFTERNOON SESSION.

Was occupied by addresses from Drs. J. H. Foster and E. Townsend, to which the public were invited.

---

#### EVENING SESSION.

Dr. R. Arthur read a report on American Dental Literature.

Dr. Townsend, before reading a paper, said :—I must premise that this paper, which I am about to read, was prepared under the expectation that subjects would be needed for discussion, and as this is an important subject, and one in which there has been a great deal of mal-practice, I thought it proper to be brought before the Society.

[Reads a paper prepared by himself on the "Treatment of Irregularities of the Teeth."]

Dr. Allen.—I must coincide with Dr Townsend with reference to the impropriety of removing the temporary teeth. I have for some time stood alone in our city in reference to extracting them. I have again and again refused to do it on the ground that it would cause an absorption of the alveoli, and a consequent contraction, and I have made it a point never to remove them unless the permanent teeth were making their appearance, and the fangs of the temporary teeth are likely to cut through the alveoli and gum. There are cases where the fangs do not absorb properly; in such cases it is better to remove them, for the permanent teeth are beginning to crowd them out. There has been a great deal of mal-practice in our city in that particular, and I am glad that the subject has come up, for it has perplexed me very much in getting the teeth straight, and I am in hopes that there are those here who have found less difficulty in regulating these teeth than I have. I have found it to take a year and a half to get a set of teeth true. My course of treatment has usually been, either to employ an inclined plane, to bring them out, or springs, or wedges or gum-elastic between the teeth or ligatures. I have adopted them all but I hope I shall get light on the subject. I think it a very important operation to be able to treat the permanent teeth successfully so as to get a true arch, but I always make it a point never to remove a temporary tooth so long as it can be retained with propriety in the socket.

To a question as to his mode of treatment, Dr. Townsend said, the cuspidati were entirely outside of the arch, the bicuspids were contracted so much that the point of the tongue could be got between them, and by putting in wooden wedges I got them so very much apart that the cuspis of the upper fell into the cuspis of the under jaw. The next thing was to bring out the incisors, that I did by making a gold band which pressed upon each of the cuspidati; then I had loops of gum elastic which were hitched upon this piece of gold, and these bands were changed every two or three days. When these were brought entirely into their places, there was another difficulty, which was this, that the molar teeth were so long that these did not strike up to the top, so that they had to be retained by ligatures. I had to tie the strings and leave them there until they could tighten them themselves.

Dr. Westcott.—I suppose this is one of those subjects which is very difficult for reporters to make clear, or to be understood by ourselves without models. There are a few general principals which may be set forth in a general way. I would simply say that it is a subject to which I have given much more attention the last two years than in all my course of practice before, and if we could have a few models, to show precisely the mode of operation, I think we could make it very interesting to ourselves. The Dr. has mentioned the use of gum-elastic in regulating teeth. There are only two general points that I think of, that I care to state, and the one is that my experience has led me to this practice. I almost invariably make the plate precisely as if I was to make artificial teeth.—Such a fixture as may be removed as easily as any plate of that sort, and with the strict injunction that it must be removed regularly and the mouth cleansed. Almost anything you can put on is liable to injure the mouth and render the breath fœtid.—And it should be easily removed, so that any intelligent person can adopt it from day to day. I can only speak of gum-elastic fixtures in a general way. I have excluded them from my practice by having something to which all the fixtures are attached, so arranged, that you can change their direction, and by means of this I have been able to regulate any cases that I have had occasion to regulate, for two or three years. An objection to gum elastic is, that if you use it of sufficient thickness it is bungling. I am only speaking of my own practice.

As I said before I have long since ceased to condemn processes. I had a case under my hand, from a distance; a little girl ten or eleven years of age came fifty or sixty miles to have a tooth regulated—a lateral incisor had come apparently almost out of the roof of the mouth, and it was behind the regular arch of the under jaw. My plan was at once to make a plate as though I intended to set in an artificial tooth, with firm clasps, to the temporary molar which she had in her mouth, then by soldering a spring on the outside of the clasp as it passed around the tooth, so that by bending it in any direction you pressed it down, and attaching the ligature to the tooth, I was enabled to throw it in any direction, that kept up a continual pressure, and it could be removed at pleasure. After straightening it a little in that way I ceased to use ligatures. I made a constant spring under the tooth, and it was so arranged that you could push it in different directions. It is necessary under such circumstances to keep the teeth apart, and instead of resorting to ivory blocks and that sort of thing, I soldered on gold blocks, and by this arrangement I was not under the necessity of seeing the case more than four or five times until its completion. After two or three days I sent her home, and she was there a fortnight, then she came to me again, and as soon as the tooth came out a sufficient distance for the under teeth to come under it, I took away the fixtures. My object in rising was mainly to express one or two general ideas. First, the necessity of having a permanent arrangement that sits easy, and which you can rely upon as a base work all the way through, and second, so arranged that it can be easily removed. By avoiding gum-elastic, and having the permanent plate so arranged, it would save a great deal of trouble.

Dr. Townsend.—For the mode of using the gum-elastic, I am indebted to Dr. Tucker, of Boston, who furnished me with some in tubes. I would state that we are very seldom compensated for this kind of work.

After this case was over to which I referred, the mother requested that I would send in my bill, which I did for $150, and the same afternoon the father called upon me with a check for $300, and said he was only too thankful that I had succeeded at all.

Dr. White.—I am very glad that this subject has been broached this evening. I think that although the regulation of the permanent teeth is a very important subject, yet it is more important to know how to preserve the temporary teeth so long that it may not be needed. It is strange that there is so much necessity for regulating the permanent teeth for the last few years. It argues either a want of confidence in nature to produce in a regular manner, or that the intermeddling of dentists have done more harm than they can remedy.

I would state, that I consider the subject of the regulation of the teeth of too much importance to discuss here without models. The President saw a contrivance in my office some years ago, which consisted of a spring, so arranged, that in its effort to straighten, all the time was pressing the teeth out. It acts in this way and does not excite inflammation. A small cut of it was given in the News Letter some time ago. The question has been asked of me here, what can we do when a child comes crying with the toothache. I would remark upon this that it is no more necessary to extract deciduous than permanent teeth. The treatment of the nerve will depend upon the condition of the tooth. It is true that as long as the tooth is in a vital condition, the fangs will go on absorbing, and as soon as the vitality ceases, the absorption will cease—maintaining the vitality of the deciduous teeth as being a means of absorption. If a patient comes to me at two or three or four years of age, I have every confidence that I can treat it as well as a permanent tooth. If the pulp is exposed, it can be destroyed and removed, and the tooth filled, and the tooth may be saved as long as required.

In reference to the treatment of toothache in children—I have less trouble in treating it, than in grown persons. It seems to me that they suffer less from the same kind of inflammation.—It is a common thing for children to cry for three or four days with toothache, till the family is out of patience, and want the tooth pulled. The tooth is tender to the touch, and the pulp is dead. I have found that by merely opening the dental cavity and getting rid of the dead nerve, the pain will subside. Every day I have to open cavities to relieve patients, and often I put nothing in at all, letting them take the risk of aching again.—When I treat the teeth for pain, I sometimes apply tannic acid and morphia to relieve the pain. In this way I coax along the deciduous teeth. I would state that I have only seen three or four cases that I can trace to natural irregularity. It is mostly artificial.

Dr. Dunning.—I wish to say that my practice with reference to deciduous teeth that are aching, corresponds almost entirely with that of Drs. White and Townsend. I am as anxious to retain them in their places as they are. There is one point, however, which I think was not touched upon by them. I think that where the pulp has died from exposure, and I have removed it from the tooth and filled the fang, I have found that all the symptoms of ulceration and irritation in the gums, have yielded much more kindly to that cleansing treatment, than in adult teeth. I found on examining my daughter's teeth, that one of them had the nerve exposed. I removed the nerve immediately, and filled its place with gold. The child has never had a moment's pain with it since. It has been with me a question I have hardly known how to decide, what was my duty when those cases were brought to me in the hurry of ordidary professional engagements—parents saying they are tired out with the crying of their children. I am perfectly satisfied that if I had the time to devote to these cases, I could generally relieve the pain and preserve the tooth till it was time to be removed. My course is generally to advise that they should retain it as long as possible. In those cases where children are brought to me regularly, I plug the deciduous teeth carefully, watching them with the same care that I do the permanent teeth. I direct the parents to have them thoroughly cleansed, not only with the brush regularly, but with floss silk, to prevent decay between them. In such cases, and with children of my own family, I have never found any difficulty; but parents do not think that children's teeth require attention. They attach about half-price degree importance to them in every respect.—

I have thought that I should like to confine my practice to the treatment of children's teeth, for there is a pleasure in knowing that you may do so much to render a long life happy.

Dr. Hill.—I would wish to say that this subject is exceedingly interesting to me, and it is important to the profession and to the public; and if any thing that we can say or do will have a tendency to spread abroad in the community, an impression of its importance, we will have done ourselves and the profession and the public generally a very important service.—There are so many difficulties in following out the dictates of our own judgment in matters of this kind, that this subject becomes more perplexing to the operator than almost any other.—In the first place, the want of a proper remuneration for the trouble.

Secondly, the difficulty of operating upon little children. It is almost impossible to get children to consent in my practice, and I have supposed that I could manage children as well as the generality of gentlemen in the profession. The great thing is in the treatment of deciduous teeth. The mere cleansing of a cavity, when there is pain, I have found to give relief. If this will not answer, I have found that the application of chloric ether is almost always successful, and it being seldom the case that I can fill a tooth in a child to give satisfaction, I nave recourse to one thing. The gentlemen will excuse me for naming it, but as we are giving the results of our experience, I may mention, that after cleansing the cavity, the easiest and best thing I know is to put in a little of "Hill's Stopping" and press it in the cavity; it has almost always given relief.

On motion, adjourned.

---

THURSDAY MORNING, AUGUST 7.

The committee appointed to revise and alter the Constitution reported progress, and were continued, to report at the next annual meeting.

Dr. E. Townsend then presented the following, as an amendment to the Constitution:

" Each and every member of this society shall either have become such by virtue of his attendance, in person, by proxy, or by letter, at the time of its formation, or shall be afterwards elected as prescribed by the Constitution, and subscribe to the same.

*Resolved*, That all delegates appointed to represent recognized Dental Colleges and Associations of Dental practitioners, organized for scientific purposes, shall be admitted to all the rights and privileges of active members of this Society, as fully as if they had been elected to such membership by ballot, under the provision of Article 6 of the Constitution."

This resolution was followed up by the following argument on the part of Dr. Townsend, which, together with the resolution, was laid on the table and ordered to be printed:

*Mr. President:*—I have offered this amendment to our Constitution for the purpose of supplying a provision which is so completely within its scope and purpose, that its absence seems a mere oversight in the original draft of the instrument. The election by ballot, which is the prescribed mode of admission to membership in all cases, as the Constitution now stands, is, of course, but a method of ascertaining the worthiness of the applicant, who, in all cases except those contemplated by the amendment now submitted, comes to us without other warranty than his personal or individual character, upon which it is right and necessary that this Society should make up a deliberate judgment before admitting him and thereby endorsing him. But delegates from recognized Dental Colleges and Associations—in other words, bodies of our professional brethren which we acknowledge and respect—come to us with credentials which we are bound to honor as much as we ask other bodies to respect and honor our own, and it is a violation of right and propriety to make such delegates wait at our door for admission, after they have sent in their cards and letters of introduction, while we go through the form of re-judging the judgment of the men who sent them. We certainly would not willingly have our trusted and honored representatives so received by any society of equals in the world, and I know we would not send representatives to any society which held itself our superior. When a reputable association of scientific men have selected delegates to any other with whom they are willing to enter into professional reciprocities, to examine in any way or to subject to any test the worthiness of such delegates, is to disregard and discredit those who gave them their appointment. I need say no more on this point, except to add that the amendment, in effect, does nothing but allow the endorsement of respectable societies and colleges to pass unquestioned with us; and members, so admitted, will come in, subject to all the conditions and entitled to all the privileges, and none other, that attach to those admitted by our present mode of initiation.—They will stand to us precisely as if we had unanimously elected them, and it will remain with them, as with others, to sign the constitution and comply with its provisions, in order to entitle them to equal participation with us in all that pertains to full membership in our society.

Mr. President—Mere verbal or ceremonial amendments to constitutions are not worthy of the time they occupy, and I would not propose one unless I thought the *words*, which I suggest, meant *things*, and the *forms* stood for important substance. We *style* ourselves, "The *American* Society of Dental Surgeons," and our objects, as they are stated in the preamble to the constitution, are, among others, "to promote union and harmony among all respectable and well-informed Dental Surgeons; and to advance the science by free communication and interchange of sentiment, either written or verbal between members of the society both in this and other countries." Thus the name and the attitude which we take, assume to fraternize the men and centralize the movement by which our profession is to be carried forward to its destined position in the world of science; and it is incumbent upon us to adjust and address ourselves to our function by every proper means that promise to promote our aim.

The adoption and publication of this amendment in connection with our other action on kindred points, I conceive will indicate the liberal and cordial spirit which animates us, and will be received as an earnest overture to the professional brotherhood, every where, for that correspondence and co-operation which must answer to the achievement of our great design. Eleven years ago our society rose out of the crowd of professors and pretenders to dental skill, for a purpose which happily stands well accomplished now, whatever agencies may take the credit of it. I mean that it drew a line of distinction between the rabble of quacks and the men of honest pretensions, expecting that the profession would soon be found *within* its membership, and the charlatans *without;* or, at least, that by its efforts to help all other agencies, a standard would soon be established for public judgment, which would render honor to whom honor was due, and rid the profession of all the responsibility and discredit of epiricism. Now this work is well enough accomplished to answer all the ends for which it was undertaken, and the attitude of offensive and defensive war should be changed in all points, so that no prejudice or suspicion shall be allowed to remain, that we are taking more care of our character and caste than of the great interests of the profession.

Mr. President—A society is like other creations made of live materials. When it ceases to grow, it begins to die; not consciously, perhaps, but none the less certainly. The laws of the human *body* well represent the laws of human societies.—Whenever the *old* ceases to be replaced by the *new*—when the work of assimilation and the incorporation of fresh elements stops, the body, or the society, is on the turn; and decline, once begun, means death for its conclusion. Churches need revivals, and political governments, occasionally refresh themselves even with a revolution. Scientific societies also demand renewal in spirit and in *men*, but they can secure it by providing for their regular growth and constaut accommodation to the changed conditions around them by their corresponding changes within them. I know I do not mistake the feelings of this association, and I am sure I understand its objects. They are both liberal and enlightened, and they aim earnestly at the greatest advancement of our profession which they can contribute to secure.

Let us manifest, by every means within the compass of our power, that we design and desire the union, harmony and co-operative activity of every worthy worker in the cause of progress; that we have no jealousies of rank and no privileges of age to defend; that we do not wish to establish a close corporation of exclusives, but a liberal republic of dental literature and science. We might invite delegates in words of respect, and welcome or wait until they offered themselves, and then admit them to the courtesies of our annual meetings; but such men as there is any interest or honor in inviting, would not come to us as guests to receive our hospitalities. They would come, and we would receive them, as equal members, on the level platform of a broad brotherhood that exchanges honors and benefits evenly.

There are Dental Colleges and Dental Societies now in several regions of our own country; and there are some associations abroad whom we would warmly welcome into friendly correspondence and fellowship of effort, and we will have done our duty in the matter, by opening our doors widely for their admission.

The fifteenth Article of the Constitution requires a majority of three-fourths of all the active members or Fellows present to carry an amendment. I am flattering myself, sir, with the hope of an unanimous vote for this one which I have the honor to propose.

Dr. Dunning's aphorisms on the treatment of exposed dental nerves, on motion of Dr. Arthur, was amended, so that in place of the first and second aphorism, it shall read—

1st. A tooth, whose nerve is exposed, *may* be permanently saved by entirely removing the pulp and filling the place occupied by it with gold.

---

THURSDAY AFTERNOON, AUGUST 7th, 1851.

On motion of Dr. Dunning, the discussion on the aphorisms, presented by him, was discontinued.

The President then read the following, (as amended by the Society):

1. The teeth, in a natural state, are exposed, and, from a variety of causes, are subject to decay. Cavities are formed both in the enamel and in the bony structure beneath it, which, by judicious management and careful treatment, may be stopped, so as to preserve the teeth in a perfectly sound and healthy state, not only for years, but for a long life. Adopted.

2. Gold is the best substance hitherto discovered for this valuable purpose. Adopted.

3. Tin foil may be used with safety; but it is of short duration when compared with gold, and cannot be permanently relied upon. The instances are but few when tin is used, where gold would not have answered better by skilful treatment. Adopted.

4. As the success of this delicate and most important operation on the teeth depends much more on the skill of the operator than it does on the substance used, the dentist should always be allowed all the time required to form and perfect the cavity, before the metal is inserted. Adopted.

5. The tenderness or soreness of the tooth, or adjacent parts, should never be permitted to operate as an apology, on the part of the patient, for restricting the operator in the preparation of the cavity, and in the use of the force necessary to the proper insertion of the filling, which should be made as hard as possible and as imprevious to moisture as solid metal. Adopted.

6. The proper introduction of the gold, in many instances, requires that a portion of the sound part of a tooth should be removed by cutting or filing, which patients frequently object to having done, from the great injury that has followed the operations of unskilful dentists. The objection should never be made if a skilful operator is employed; if not, let the teeth remain until such an one can be found, for the former will do them, in the simplest operation, much greater injury than benefit. Adopted.

Dr. Bridges.—I would like to get the views of the members on filing the teeth. I have had patients to say' " Why, Doctor, what do you do with those files? Dr. Parmly and others don't use them." I would like to know how much you use them?

The PRESIDENT.—Those who say that the President does not use files are mistaken.

7. It becomes necessary, in many instances, to separate teeth preparatory to stopping them, in order to afford the operator a suitable access to the cavity; and sometimes, particularly in the front teeth, instead of cutting or filing, it is desirable to effect such separation temporarily, which may be done by gently forcing between the teeth small wedges of wood or slips of India rubber. Adopted.

8. The use of amalgams and pastes which, being introduced in a soft state, become more or less hard in the tooth, is utterly prohibited by the Society which gives its authority to these maxims of dental practice—it having been proved unsuitable and highly objectionable as an ordinary filling for teeth, even, when used by the best practitioners. Adopted.

DR. WHITE.—I would like to hear that aphorism read again As it was written some years ago, we might make some alteration now. (It is read.) I would move that all after "many of those who make a wholesale use of it," be stricken out.—Carried.

DR. TOWNSEND.—I would make it a little stronger than it is at present, by saying that the article was unsuitable, *even* when used by the best practitioners.

DR. WHITE.—I am no advocate of paste or amalgam, having never used it in my life. But it seemed to me that this language was too strong for us to use, and it would cut off those young men who would like to have the privilege of making experiments which would satisfy them.

PRESIDENT.—I would make this alteration: "It having been proved unsuitable and highly objectionable as an ordinary filling for teeth, even when used by the best operators among those who advocate its use." Carried.

9. Some teeth, in their very structure, are more liable to decay than others; and, whatever may be the cause of their liability, whether hereditary or superinduced by disease of the general system, or by local affections, it becomes exceedingly difficult to save them. Therefore, the best material and the best skill should always, and in all such cases, be employed.—Adopted.

10. The greatest difficulty connected with stopping in such a manner as to arrest decay entirely and save the teeth, results from the habit of individuals, in postponing the operation until the decay has reached the internal or nerve cavity, or has otherwise destroyed so much of the substance of the tooth as to make stopping unadvisable or impracticable. Adopted.

Dr. Arthur.—It seems to me that the latter clause of the aphorism changes its meaning. It is at least ambiguous, if you regard the destruction of the nerve as an useful operation. It seems to me that this would lead a person, unacquainted with the subject, to the conclusion that, if the nerve became exposed it would be impossible to save the tooth.

Dr. Robertson.—I concur with Dr. Arthur in that, and I think if the word "sometimes" were put in, it would be an advantage.

President.—I will alter it by putting in "in many instances."

11. A cavity is never too small to require a filling, provided there be a positive perforation of the enamel, and more especially if the cavity extends into the bony structure beneath.—Adopted.

12. As stopping teeth is the best and most satisfactory of all the operations performed by the dentist, no substance but gold should be relied upon as a permanent filling, and no skill but the best should be employed in using, adapting and securing it. Adopted.

Dr. Arthur.—As it was supposed these aphorisms would give rise to discussion, and as they have not, I would propose that we discuss the subject of filling the teeth generally; and that we defer the discussion on the irregularity of the teeth till next year, and that in the meantime, every gentleman so disposed, take this subject into consideration, and if he can bring models and cases with him and state to the society his mode of treatment, I think that a great deal of good might be accomplished—for this is a subject about which we have no general views. Every one has pursued his own course, without reference to any body else.

Dr. White.—If I am in order, an idea strikes me with reference to one of the aphorisms read, that I would like to bring before the Society. I think it would be well to discuss the subject of what condition should the gold be in, to be best suited for filling cavities completely.

Dr. Bridges.—Before this matter in relation to the regulation of the teeth is passed, I would like to make one or two remarks. Last night we had to choke off this subject, and I had hoped that this morning we would have an opportunity of communicating freely on this subject.

Dr. Arthur.—I made a motion that the discussion on this subject be deferred till the next meeting.

Dr. Bridges.—I merely wanted to make an observation that the members should go to asylums, in order to see the condition of children's teeth.

Dr. White moves that the Society go into a discussion of the best condition of the gold for plugging teeth.

Dr. Hill.—Would it not be better to make a motion that we now have an opportunity for miscellaneous remarks, and I now make that motion. It is carried.

Dr. Robertson.—If there is nothing else before the Society I would state that hitherto in my practice, I have taken some pains to find what would make the best dies for striking up plates. Latterly, I use an alloy by which I expect to fit the first time, and if I do not, I am responsible for it. The alloy is a mixture of one part copper, two parts antimony, and six parts tin.

The copper is to be first melted. The advantages of it are that it shrinks very slightly, and it is so hard that the minutest point on it cuts its mark in a gold plate. I use lead for the female casting, and sometimes I prefer using type metal. Pure lead, without considerable care, may stick, and it may be necessary then to use chalk. It is so hard that it has a disadvantage of making the inside of the plate a little rough.

Dr. Bridges.—I merely wanted to remark that I have been for some time dentist extraordinary, ordinary and certainly general, to the King's county poor-house. It is very easy for a man to get a theory, but it is very hard to find one to suit all cases.

With the practice of Drs. White and Parmly, it is very different from what it is in a place where they have perhaps never seen a dentist in their lives. I can find five deformities from leaving the deciduous teeth in too long, for every one that a stated practitioner can show me from taking them out too soon. I had an invitation a few years ago to visit two asylums, and I found the children's teeth in a most wretched condition. Some of the deciduous teeth were as tight as the permanent, that were crowding others out of their places. I think the deciduous teeth should in all cases be taken out when the child becomes eleven or twelve years of age. I have often waited for the expansion of the maxillary bone, and have often been disappointed. The request I was going to make, was that there be a committee of an indefinite number appointed to investigate this matter of children's teeth, and that they take the opportunity, between this and the next meeting, of visiting asylums, where the children have not been visited by dentists, and that they supply casts at the next meeting.

This proposition was adopted, and the committee appointed.

Dr. White.—I offer the following resolution, with a view of establishing another committee upon a subject that I deem quite as important as this.

*Resolved*, That a committee be appointed to make microscopic observations, with particular reference to the characteristics of salivary culculus, and the fluids of the mouth in connection with the human teeth, and report at the next annual meeting.

The President.—This is a very important subject. Dr. Goadby, who assisted in the investigations made by Dr. Nasmyth, of Edinburgh, is now in this country, and I have no doubt will give us such information as will make these investigations most useful.

The resolution was adopted, and the following gentlemen were appointed the committee: J. D. White and E. Townsend, of Philadelphia.

Dr. White.—I wish to call special attention to the manner in which Dr. Arthur uses gold for filling the teeth.

Dr. Arthur.—I have for some time been making experiments with gold for filling teeth, and have fallen upon what I regard a very great improvement. I have no doubt gentlemen will be very much surprised at it, and will be doubtful of its utility. I use gold never finer than No. 30, and from that to No. 50, but I generally rely upon No. 30. I use this not only for strong but for frail and most delicate cavities. By using this, I can do business about one-third greater in amount, and not only as well as when I used finer gold, but a great deal better. This is well worth the trial, and can be easily tested. The manner in which it is used is essential to success.

It is well known that No. 15 has been found very harsh and unmanageable, and so it is if the gold I use is folded even once. I take simply a slip of the gold, nearly or quite the size of the cavity, and I generally use very small instruments for condensing after the gold is introduced. I don't know, however, that I have made any material change in my instruments since I commenced to use this thicker gold. I have found gold of this kind, in my hands, more manageable than any other.—It is obvious that the surface of the filling with this gold must be better—there must be a fewer number of edges, and it will take a better polish.

President.—I don't know whether any one is present who was at a meeting at which I spoke of the gold used by Waite, of London. He was the Hudson of England when I first resided there. I thought the stoppings from his hand were different from others, and I felt anxious to know how he made them so solid; I made all the inquiries I could outside of his house, then I went to his house, and his brother brought into the room where I was, a part of a sheet of gold which was about the thickness of the lead which lines the China tea chests. He said there was but one man in London that could prepare gold in that way—it was as soft as the lead itself, and I have often wished that I could find such, but I never could. This corresponds with the remark of Dr. Arthur. Some years ago a very ingenious man who had something to do with the invention of the cotton gin, took it into his head that he could do much in the way of saving teeth. He drilled into the tooth then cut a screw on the end of a gold wire, and screwed it into the tooth and cut it down, and it was one of the finest fillings I have ever seen.

Dr. Arthur.—I have used gold from several manufacturers. That which I now use I get from Abbey & Son. I find that the gold produced by them is generally of uniform quality at least. I do not wish to make an invidious comparison between the gold made at this establishment and that of other manufacturers; but it is of great importance in using gold of this kind that it should be of the best quality, and perfectly annealed.—I would certainly advise any one who is disposed to use the gold of the kind I propose, if they are not satisfied on the first trial, to use that of several manufacturers before they throw it aside.

Dr. Allen.—I have invariably used gold as high as No. 12, and prefer it, but I am the only one that uses it in our city.

Dr. White.—I have used gold that is very heavy, and very light, and where we had to double the sheet upon itself, I am sure all must admit that it must leave a little opening and must become a little hard, so that it takes more pressure to accommodate it to the cavity—hence in a great many thin cavities I have used No. 3.

I will cite the treatment of a case in point. A lady had a front incisor filled three or four times that was decayed from the outer edge, leaving nothing standing except the enamel posterially and at the cutting edge. It had been filled several times in three months, but carefully, for fear of breaking. The last dentist, a gentleman in practice from twenty to twenty-five years, told her it was impossible to fill it without breaking.—Some one induced her to call upon me, and I had some of this thin gold which I put into the cavity with gentle pressure.—Having removed the decay the cavity was larger than before. The plan I took to cleanse it was one suggested by Dr. Williams. After removing the principal part of the decay, I worked in with a small instrument and cotton, then I put this fine gold in until the cavity was filled, until I came within the thickness of the enamel to its surface. There was only now a small cavity and this I completed with No. 4. The lady is now a resident of your city. She was absent one year, and upon examination, the tooth appeared as perfect as on the day she left. I merely state the case to show that very light gold is sufficient to accomplish the end desired; but whether heavy gold would have accomplished the same end I know not. I have tried No. 50 in a few similar cases, and I believe I made very respectable fillings, but not so good as with gold that I was accustomed to.

Dr. Arthur.—I have found that No. 30 worked a great deal better that No. 50, so much so that I have discarded it.

Dr. Westcott.—If this discussion is at a point where an interruption may be tolerated, I would like to offer a resolution that when this Society adjourn, it adjourns to meet at Syracuse New York.

Dr. Hawes wished to amend the resolution; in the place of Syracuse, insert Newport.

Dr. Allen wished to amend by inserting Cincinnati.

The further discussion of this resolution was postponed.

Dr. Dunning.—I generally use No. 4, and would like to get No. 3 if I could.

Dr. Arthur.—It must be remembered that the point on which the principal advantage depends, is that it must be used in a single strip and not doubled upon itself.

Dr. White.—Is it not being doubled while being put into the cavity. When you put the gold into the cavity where you want it, does it remain there?

Dr. Arthur.—It is not a half dozen or more surfaces folded together to be condensed afterward, but is condensed, fold after fold, as it is put into the cavity to be filled. But the utility of gold in this form can only be tested by actual trial.

Dr. Westcott.—On the point of keeping the filling where you put it, I will make a single remark. I have been much in the habit, for two or three years, of filling teeth with both hands at once. I had never done it or seen it done before. I manage to hold my napkins in a way that I can fill with my left hand and use two instruments, where the cavity is of such a shape that it is important that you have one point packed thoroughly so as not to have it come out again. It is very risky, if you have a piece tumble out several times. You get it hard, by the working. I use only one hand for filling, and the other for holding it precisely and firmly where I know it will be wanted. I think that every dentist will find that he can do more in that way than he can imagine. This is not one of those cases where you are under the necessity of hiding from your left hand what your right hand doeth. When there are large cavities, I uniformly employ an assistant. That idea of keeping the foil where you put it is important, with bicuspides particularly, and those teeth where the cavity is long and narrow.

Dr. Foster.—I invariably use No. 4 gold foil in all cases; large as well as small cavities. In some cases I find it extremely difficult, even with most malleable foil, to keep it where I place it. If I have to use a different foil, I shall have to learn my lesson over again; for, as I use it, it must be rolled together and it is important that I should have the most malleable foil. I have tried, repeatedly, in large cavities, to use thicker foil, thinking I might get along faster; but it rolls about and gets out; and I now invariably use No. 4.

Dr. J. Tucker, of Boston.—I have used nothing but No. 4 for the last fifteen years, except where other has been sent to me for trial; and in such cases I have always failed, and given it up.

Dr. Arthur.—The kind of gold I have recommended could not be used in the manner described by Dr. Foster.

Dr. White.—I would like to state in what manner I use it. For general use, I take a strip of gold and roll it together spirally. I use No. 4 and No. 6, pretty equally divided. If I can deliberately press upon the tooth, I use No. 6, but in lateral cavities I use No. 4. If the cavities are very large I use pellets. I take a straight and narrow watch spring, to which I have added a little round piece of wire, so that I can turn it with facility; I fold it on this watch-spring flat; this facilitates my rolling. In some few cases I use it in that form for small cavities. In this way, it is folded so that every leaf is in close contact, and the atmospheric air can be pressed out better than if it is folded.

Dr. Arthur.—Gentlemen may have been in the habit of using the gold of the ordinary number in a different way from what I have done. I almost invariably folded it in strips on itself. This, it will be seen, is a considerable step toward using the thicker gold, which I propose. And it may not, therefore, be found, at once, so advantageous in the hands of others as I have found it. But I am convinced that, if it is fairly tried, it will be found very useful in many cases, even if it should not be adopted for general use.

President.—The next business, if there is no more discussion, is the election of members.

When Mr. C. C. Williams of Philadelphia, was elected.

The President returned his thanks to the friends of the Society for their attendance.

Election of officers took place, when the following gentlemen were elected:—Dr. E. Parmly, of New York, President; Dr. E. Townsend, of Philadelphia, 1st Vice President; Dr. J. H. Foster, of New York, 2d Vice President; Dr. J. Tucker, of Boston, 3d Vice President; C. O. Cone, of Baltimore, Corresponding and Recording Secretary; E. J. Dunning, of New York, Treasurer; D. R. Parmly, of New York, Librarian. C. O. Cone, E. J. Dunning, D. R. Parmly, Publishing Committee. J. H. Foster, E. J. Dunning, E. Parmly, A. C. Hawes, C. A. Harris, H. W. Fenn, Executive Council and Examining Committee.

Dr. R. Arthur was chosen to deliver the opening address at the next annual meeting. The following members were appointed to prepare essays to be read at the next annual meeting of the Society, on some subject connected with dental theory or practice: Drs. J. D. White, E. Townsend, E. G. Tucker, A. Robertson, John Allen.

On motion, the Committee on American Dental Literature was discharged from further duty.

On motion, a Committee was appointed to report, at the next annual meeting, on the state of Foreign Dental Literature. Dr. C. A. Harris was appointed that Committee.

Adjourned to meet at Newport, R. I., on the first Tuesday of August, 1852.

